# Leukoaraiosis, a Common Brain Magnetic Resonance Imaging Finding, as a Predictor of Traffic Crashes

**DOI:** 10.1371/journal.pone.0057255

**Published:** 2013-02-20

**Authors:** Kaechang Park, Yoshinori Nakagawa, Yasuhiko Kumagai, Mitsuhiro Nagahara

**Affiliations:** 1 Traffic Medicine Laboratory, Research Organization for Regional Alliance, Kochi University of Technology, Kami-shi, Kochi, Japan; 2 Department of Management, Research Organization for Regional Alliance, Kochi University of Technology, Kami-shi, Kochi, Japan; 3 Regional ITS Social Laboratory, Research Organization for Regional Alliance, Kochi University of Technology, Kami-shi, Kochi, Japan; Charité University Medicine Berlin, Germany

## Abstract

**Background:**

There are no reported studies on the relationship between traffic crashes and brain tissue changes in healthy drivers. The relationship between traffic crashes and leukoaraiosis, a common magnetic resonance imaging finding, was investigated in this study.

**Methods:**

A total of 3,930 automobile drivers (2,037 men and 1,893 women; age, 21–87 years) who underwent brain magnetic resonance imaging as part of total health check-ups and answered a road traffic questionnaire were examined to determine whether asymptomatic leukoaraiosis was associated with various types of traffic crashes. Multiple logistic regression analysis was performed to elucidate the relationship between leukoaraiosis and various types of traffic crashes.

**Results:**

Subcortical leukoaraiosis was diagnosed in 28.52% of all subjects, whereas periventricular leukoaraiosis was diagnosed in 9.57% of all subjects. Adjusted odds ratios for involvement in all types of traffic crashes were not significant for subjects with periventricular leukoaraiosis; however, they were significant for subjects with multiple and large multiple subcortical leukoaraiosis. Adjusted odds ratios for involvement in crashes at crossroads were 1.09 (95% confidence interval [CI], 0.60–2.00) for subjects with single subcortical leukoaraiosis, 3.35 (95% CI, 2.36–4.77) for subjects with multiple subcortical leukoaraiosis, and 2.45 (95% CI, 2.36–4.98) for subjects with large multiple subcortical leukoaraiosis. Periventricular leukoaraiosis was not significantly associated with crossroad crashes. Involvement in crashes of any type, parking lot crashes, and rear-end collisions showed no significant association with either subcortical or periventricular leukoaraiosis.

**Conclusions:**

Multiple subcortical leukoaraiosis, but not periventricular leukoaraiosis, is significantly associated with traffic crashes, in particular, crossroad crashes. This association is independent of sex, age, and driving exposure. To our knowledge, this is the first evidence describing the relationship between brain tissue changes and traffic crashes.

## Introduction

The World Health Organization has shown that road traffic accidents are a huge concern in the field of public health and development because they kill approximately 1.3 million people every year and injure or disable 20 to 50 million more worldwide. Without appropriate action, these injuries will rise dramatically by the year 2020, particularly in countries that are rapidly increasing their use of motorized vehicles [1], [2]. Road traffic injuries are predicted to hold the fifth highest rank in terms of annual disability adjusted life years (DALYs) in 2030 [1]. The socioeconomic impact of these injuries on individuals, families, and communities has remained enormous. Therefore, there is an urgent need for fundamental measures directed toward the prevention of road traffic injuries. Nevertheless, conventional measures have been exclusively focused on vehicle-related, infrastructural, and environmental factors or factors affecting the vulnerability of the human body, such as tiredness, drowsiness, and limited visibility [1], [2]. Human factors that cause traffic accidents include not only vulnerabilities but also the predictive and executive functions of the brain. These brain functions are affected by aging and factors causing brain tissue damage, i.e., space-occupying lesions such as tumors and hematomas or ischemic lesions such as infarcts and leukoaraiosis (LA). The term LA is derived from the Greek terms *leuko* (white) and *araiosis* (rarefaction) and is currently recognized by the presence of different histopathological changes such as vacuolization (spongiolosis), demyelination, loss or gain of glial cells, apoptosis of oligodendrocytes, and Wallerian degeneration [3], [4]. LA is also characterized by white matter hyperintensity on T2-weighted magnetic resonance imaging (MRI) and is classified as subcortical LA (SLA) and periventricular LA (PLA) according to the site of involvement [5]. The risk factors for both SLA and PLA are considered to be advanced age, smoking, atherosclerotic diseases such as hypertension and hyperlipidemia, diabetes, and metabolic syndrome [6], [7]. In recent years, remarkable advancements in MRI equipment, software, and imaging procedures, including the combined use of T1-weighted, T2-weighted, and fluid-attenuated inversion recovery (FLAIR) images, have enabled more accurate diagnosis of LA, especially a single small spot of LA, which is an earlier stage of LA progression [8]. Approximately 30% of apparently healthy individuals reportedly have LA, including the minimum type. These are usually diagnosed through brain MRI check-ups, which have become uniquely prevalent in Japan [6], [7]. LA is a common type of change in brain tissue, and many drivers who operate automobiles are unaware of its existence. There have been many longitudinal population-based studies conducted mainly in the elderly, and these studies have reported associations between severity and/or progression of LA and dementia, cognitive impairments, and executive dysfunctions such as decline in processing speed, particularly in cases of PLA [9]–[12]. However, minimum or mild LA, generally diagnosed as asymptomatic, has not been adequately characterized because a majority of apparently healthy individuals have not undergone MRI examinations. In this study, we used brain MRI check-ups to determine whether asymptomatic LA in ordinary and healthy drivers was associated with traffic crashes.

## Methods

### Data

Data for this study were collected from April 2010 to November 2011 from the Brain Checkup Center (BCC) that is affiliated with Kochi University of Technology, Kochi Prefecture, Japan. We examined 3930 healthy subjects (2037 men, 1893 women; age, 21–87 years; average age, 52.7 years; median age, 51 years) with no history of stroke or cerebral diseases, including asymptomatic lacunar infarction. All subjects lived and drove automobiles in Kochi Prefecture, visited BCC, and underwent brain MRI as part of their routine health check-ups and answered a questionnaire on road traffic crash involvement during the past 10 years. Detailed information on driving frequency and traffic crashes experienced during the past 10 years was gathered from personal interviews conducted at BCC by all participating researchers except the author (KP). Driving exposure was classified into 4 categories according to total driving hours per week: 1, ≤2 h per week; 2, ≤5 h per week, 3, ≤10 h per week; and 4, ≥10 h per week. The types of traffic crashes were classified into 4 categories: 1, all types; 2, crashes in parking lots; 3, crashes at crossroads (crashed vehicles including motor bicycles); and 4, rear-end collisions (nonresponsible cases were excluded). The term healthy was defined as the absence of symptoms or gross neurological deficits before and at the time of enrollment in the study. All subjects satisfied this criterion and provided written informed consent for participation in the study. This study was approved by the institutional review board at Kochi University of Technology.

### MRI Examinations

A 1.5-T MRI system (ECHELON Vega; Hitachi Medical Corporation, Tokyo, Japan) was used to perform MRI examinations. The imaging protocol included T2-weighted spin-echo [repetition time/echo time (TR/TE) = 5800/96 ms], T1-weighted spin-echo (TR/TE = 520/14 ms), and FLAIR (TR/TE = 8500/96 ms; inversion time = 2100 ms) images. LA was defined as a focal lesion ≥2 mm in diameter, with hyperintensity on T2-weighted and FLAIR images and without prominent hypointensity on T1-weighted images. The thickness of MRI slices was 4 mm, and 3 sections of the axial, sagittal, and coronal 2D views were used for LA diagnosis. SLA was defined as the presence of hyperintensity in the subcortical white matter (SWM; none), a single dot in the SWM (single), multiple dots in the SWM (multiple), or multiple patches in the SWM (large multiple). PLA was defined as the absence of hyperintensity surrounding the ventricles (none) and the presence of periventricular hyperintensity from the rim to the confluence (positive).

**Figure 1:**
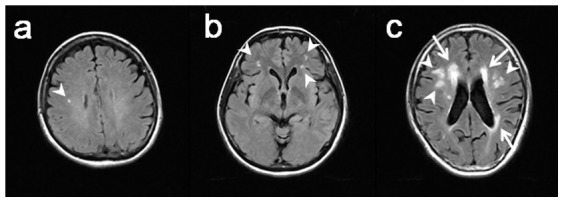
Fluid-attenuated inversion recovery (FLAIR) images of leukoaraiosis. FLAIR images of single subcortical leukoaraiosis (SLA) (a), multiple SLA (b), large multiple SLA (c), and periventricular leukoaraiosis (PLA) (c). Arrow heads and arrows show SLA and PLA, respectively.

Figure 1 shows typical cases of SLA and PLA. Two trained neurosurgeons (KP, HM) who were blinded to the crash involvement data assessed the presence of LA on MRI. When the 2 investigators had differing opinions, the SLA and PLA classifications were determined by consensus.

### Statistical Analysis

Multiple logistic regression analysis was applied to examine whether LA was a significant predictor of involvement in (i) crashes of any type, (ii) crashes at parking lots, (iii) crashes at crossroads, and (iv) rear-end collisions. In analysis (i), the objective variable was defined by allocating a value of 1 to the respondents who had caused at least 1 crash in the last 10 years and a value of 0 otherwise. In analyses (ii) to (iv), a value of 1 was allocated to the respondents who had encountered crashes of type (ii), (iii), or (iv) in the last 10 years. For each analysis, LA was included in the model in 2 ways. This means that a total of 8 (4 multiplied by 2) models were constructed. In the first way, 3 dummy variables that took values of 1 when SLA was either the single, multiple, or large multiple type were defined. In the second way, 1 dummy variable that took a value of 1 when PLA was positive was defined.

In each of the 8 models, sex, age, and driving exposure were also included as explanatory variables. Values of sex, age, and driving exposure were categorized into 2, 5, and 4 classes; therefore, 1, 4, and 3 dummy variables, respectively, were defined and included in the models. The program was written in Mathematica version 6.0 (Wolfram Research, Inc., Champaign, IL, USA), referring to the study by Agresti (1999) [9].

## Results

**Figure 2:**
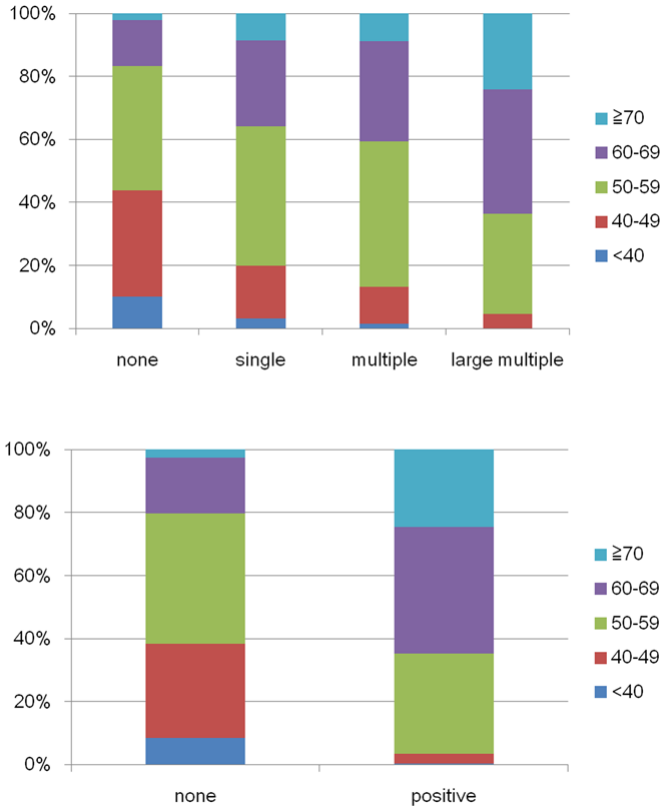
Percentage distribution of leukoaraiosis according to age. Upper panel) Percentage distribution of the various types of subcortical leukoaraiosis according to age. Lower panel) Percentage distribution of the various types of periventricular leukoaraiosis according to age.

In this study, 2676 subjects were aged between 40 and 59 years, indicating that middle-aged individuals formed a majority of the subjects (Tables 1 and 2). The frequencies of SLA and PLA were 28.52% and 9.57% of the total, respectively (Tables 1 and 2). According to the severity of SLA, the ratio of elderly subjects >60 years increased whereas that of subjects <49 years decreased (Figure 2 upper panel). More than half the subjects with PLA were >60 years, whereas approximately 80% subjects without PLA were <59 years (Figure 2 lower panel). One hundred fifty-three subjects had been involved in parking lot crashes, 177 in crossroad crashes, and 148 in rear-end collisions (Tables 1 and 2). Any types of traffic crashes accounted for 162 cases, which occurred from speeding on curved roads, improper lane changes on straight roads, improper departure from parking lots, failure to follow the right way, and adverse weather conditions (e.g., rain, snow, fog). These subjects formed small groups; therefore, we did not investigate the association between LA and crash involvement for these individual types.

**Table 1:** Adjusted odds ratios for various types of traffic crashes derived using explanatory variables of sex, age, subcortical leukoaraiosis (SLA), and driving exposure.

	Traffic Crashes of All Types		Crashes at Parking Lots		Crashes at Crossroads		Rear-End Collisions	
Explanatory Variables	Cases/Participants	Adjusted OR (95% CI)	Cases/Participants	Adjusted OR (95% CI)	Cases/Participants	Adjusted OR (95% CI)	Cases/Participants	Adjusted OR (95% CI)
Gender								
Female	312/1893	1 [Reference]	66/1893	1 [Reference]	90/1893	1 [Reference]	69/1893	1 [Reference]
Male	333/2037	0.97 (0.81–1.16)	87/2037	1.21 (0.87–1.69)	87/2037	0.84 (0.62–1.15)	79/2037	1.05 (0.75–1.47)
Age								
<40	65/303	1 [Reference]	17/303	1 [Reference]	13/303	1 [Reference]	17/303	1 [Reference]
40–49	190/1079	0.76 (0.56–1.04)	40/1079	0.64 (0.36–1.16)	47/1079	0.93 (0.50–1.75)	45/1079	0.74 (0.42–1.31)
50–59	253/1597	0.62 (0.45–0.85)	60/1597	0.65 (0.37–1.15)	78/1597	0.84 (0.45–1.53)	54/1597	0.61 (0.34–1.07)
60–69	106/766	0.51 (0.36–0.72)	29/766	0.65 (0.35–1.22)	34/766	0.66 (0.33–1.32)	23/766	0.54 (0.28–1.06)
≥70	31/185	0.62 (0.38–1.01)	7/185	0.64 (0.25–1.65)	5/185	0.35 (0.12–1.03)	9/185	0.94 (0.58–1.54)
Subcortical leukoaraiosis								
none	434/2790	1 [Reference]	109/2790	1 [Reference]	96/2790	1 [Reference]	109/2790	1 [Reference]
single	51/354	1.01 (0.73–1.38)	13/354	0.97 (0.54–1.75)	12/354	1.09 (0.58–2.05)	12/354	0.90 (0.48–1.54)
multiple	135/638	1.67 (1.32–2.11)	25/638	1.04 (0.65–1.67)	60/638	3.35 (2.36–4.77)	22/638	0.94 (0.58–1.54
large multiple	25/148	1.32 (0.83–2.12)	6/148	1.08 (0.45–2.62)	9/148	2.46 (1.17–5.18)	5/148	0.84 (0.33–2.20)
Driving Exposure a								
1	251/1807	1 [Reference]	61/1807	1 [Reference]	67/1807	1 [Reference]	54/1807	1 [Reference]
2	160/949	1.25 (1.00–1.55)	38/949	1.17 (0.78–1.77)	42/949	1.17 (0.79–1.74)	47/949	1.70 (1.15–2.51)
3	152/766	1.54 (1.24–1.91)	32/766	1.25 (0.81–1.92)	42/766	1.51 (1.02–2.23)	30/766	1.34 (0.85–2.10)
4	82/408	1.57 (1.19–2.06)	22/408	1.58 (0.95–2.64)	26/408	1.80 (1.13–2.89)	17/408	1.40 (0.80–2.48)

Notes.

a : Frequency of driving (1 = Two hours or less per week, 2 = Five hours or less per week, 3 = 10 hours or less per week, 4 = More than 10 hours per week).

**Table 2:** Adjusted odds ratios for various types of traffic crashes derived using explanatory variables of sex, age, periventricular leukoaraiosis (PLA), and driving expos.

	Traffic Crashes of All Types		Crashes at Parking Lots		Crashes at Crossroads		Rear-End Collisions	
Explanatory Variables	Cases/Participants	Adjusted OR (95% CI)	Cases/Participants	Adjusted OR (95% CI)	Cases/Participants	Adjusted OR (95% CI)	Cases/Participants	Adjusted OR (95% CI)
Gender								
Female	312/1893	1 [Reference]	66/1893	1 [Reference]	90/1893	1 [Reference]	69/1893	1 [Reference]
Male	333/2037	0.98 (0.82–1.17)	87/2037	1.20 (0.86–1.67)	87/2037	0.86 (0.63–1.18)	79/2037	1.05 (0.75–1.47)
Age								
<40	65/303	1 [Reference]	17/303	1 [Reference]	13/303	1 [Reference]	17/303	1 [Reference]
40–49	190/1079	0.78 (0.57–1.07)	40/1079	0.65 (0.36–1.17)	47/1079	1.00 (0.53–1.87)	45/1079	0.74 (0.42–1.31)
50–59	253/1597	0.68 (0.50–0.94)	60/1597	0.66 (0.38–1.14)	78/1597	1.09 (0.60–2.01)	54/1597	0.61 (0.34–1.07)
60–69	106/766	0.59 (0.42–0.85)	29/766	0.65 (0.35–1.22)	34/766	0.96 (0.49–1.87)	23/766	0.55 (0.29–1.06)
≥70	31/185	0.76 (0.47–1.25)	7/185	0.65 (0.25–1.67)	5/185	0.53 (0.18–1.57)	9/185	1.02 (0.42–2.46)
Periventricular leukoaraiosis								
none	588/3554	1 [Reference]	138/3554	1 [Reference]	156/3554	1 [Reference]	137/3554	1 [Reference]
positive	57/376	0.98 (0.72–1.34)	15/376	1.04 (0.58–1.87)	21/376	1.49 (0.90–2.48)	11/376	0.73 (0.37–1.44)
Driving Exposure^a^								
1	251/1807	1 [Reference]	61/1807	1 [Reference]	67/1807	1 [Reference]	54/1807	1 [Reference]
2	160/949	1.26 (1.01–1.56)	38/949	1.17 (0.78–1.77)	42/949	1.20 (0.81–1.77)	47/949	1.70 (1.15–2.51)
3	152/766	1.54 (1.24–1.91)	32/766	1.25 (0.81–1.92)	42/766	1.51 (1.02–2.23)	30/766	1.32 (0.84–2.08)
4	82/408	1.55 (1.18–2.04)	22/408	1.58 (0.95–2.64)	26/408	1.79 (1.12–2.86)	17/408	1.40 (0.80–2.48)

Notes.

a : Frequency of driving (1 = Two hours or less per week, 2 = Five hours or less per week, 3 = 10 hours or less per week, 4 = More than 10 hours per week).

When LA was included in the model according to the degree of SLA, the significant predictors of involvement in any type of traffic crashes were age, driving exposure, and multiple and large multiple SLA (Table 1). With regard to involvement in crossroad crashes, the adjusted odds ratios (AORs) were 1.09 [95% confidence interval (CI), 0.60–2.00) for the subjects with single SLA, 3.35 (95% CI, 2.36–4.77) for the subjects with multiple SLA, and 2.45 (95% CI, 2.36–4.98) for the subjects with large multiple SLA (Table 1). However, parking lot crashes and rear-end collisions were not significantly associated with SLA (Table 1). In contrast, when LA was included in the model according to the degree of PLA, it was not a significant predictor of crash involvement in any of the 4 analyses (i) to (iv) (Table 2).

## Discussion

In this study, the proportion of subjects with LA (approximately 30% of the total) was relatively higher than that reported in most other studies [10]–[13], although it was almost the same as that reported in our previous study involving similar age distributions and diagnostic procedures [6]. The discrepancy between our study and other study results was probably because the majority of the subjects in our study included middle-aged and relatively young individuals. Another reason could be that smaller spots of SLA can be diagnosed from 4-mm thin image slices, 3 directions of the imaging section, and 3 combined images. A combination of all 3 images, including T1-weighted, T2-weighted, and FLAIR images, is a more efficient technique for detecting LA compared with only T1-weighted and T2-weighted images [8].

Interestingly, SLA was significantly associated with crossroad crashes but not parking lot crashes or rear-end collisions. This finding suggests the following scenario. When an individual drives more carefully at crossroads, he or she must pay attention to the right, left, forward, and backward directions for moving vehicles and pedestrians. He or she may require more attentive abilities to avoid crossroad crashes than those required to avoid parking lot crashes, which involve slowing or stopping situations, or those required to avoid rear-end collisions, which involve forward-watching situations. Global cognitive impairments, including decline of attentive abilities, have been reported to be associated with the progression of SLA [14]. Although our study was cross-sectional, SLA could involve some decline in attentive ability, resulting in significant association with crossroad crashes. However, it should also be noted that a higher degree of SLA does not necessarily imply a higher likelihood of involvement in crossroad crashes. As shown in Table 1, the AORs for crossroad crashes relatively decreased in the subjects with large multiple SLA compared with those in subjects with multiple SLA, even though large multiple SLA damages brain tissue more extensively. This may be because approximately 60% of the 129 subjects with large multiple SLA were >70 years old (data not shown). Compared with younger drivers, elderly drivers have a tendency to recognize dangerous driving and change their driving styles before causing traffic crashes. In addition, the elderly subjects in our study had higher education and socioeconomic status and were more likely to drive carefully and safely. If this hypothesis is correct, detecting LA by MRI and informing LA drivers of their condition may help prevent traffic crashes in the future.

LA, which is considered to be a form of histopathological damage to brain tissue, is supposed to cause some decline in attentive functions of the brain, which could have resulted in the higher likelihood of traffic crash involvement observed in the present study. Minimum or mild progression of LA, which accounted for a majority of the cases in this study, has seldom been elucidated. However, an autopsy study of brains from subjects with LA showed that cerebral microvascular density decreased not only in LA lesions but also in healthy appearing white matter lesions [4]. Reportedly, diffusion tensor MRI has also revealed the alternation of diffusion anisotrophy effects in the normal-appearing white matter around LA lesions [15], [16]. Microstructural changes in the underlying tissue, which are responsible for LA, may affect more extensive areas of the brain, resulting in specific types of decline in brain function. Furthermore, the severity of LA may not be proportional to the decline in brain function. Previous studies reported that, elderly drivers who are considered to be potential LA carriers had a tendency toward more fixation or shorter saccades of visual search patterns during crossroad driving simulations compared with younger drivers [17], [18]. The visual search patterns of the LA drivers in this study may have been different from those of non-LA drivers. Further investigations on whether brain functions are affected by progression of LA, particularly minimum or mild LA, are required to validate our results.

The fact that only SLA, and not PLA, was significantly involved in crossroad crashes remains an enigma. Anatomically, the periventricular region has a high density of long associating fibers connecting the cortex with subcortical nuclei and other distant brain territories [19], [20]. The characteristic architecture of the periventricular area is considered to be more vulnerable to damage compared with other white matter areas. This is because most previous studies have shown that decline in brain function is higher in patients with PLA than in patients with SLA [11], [21], [22]. In our study, 48.7% subjects with PLA had the minimum type while 39.4% had the mild type (data not shown). Minimum and mild types of PLA close to ventricular walls may alleviate the damage to long connecting fibers, thus resulting in no significant association with traffic crashes. On the other hand, the subcortical area has the high density of short-looped U-fibers connecting adjacent cortical regions [19], [20]. Assuming that SLA affecting connectivity between neighboring brain regions may be involved in temporary or fluctuating decline in brain function and that PLA affecting connectivity between distant brain regions may be involved in continuous or stable decline in brain function, it would be another phenomenon if unexpected momentary cognitive or perceptual decline, that is, the hypothetical effect of SLA, was responsible for crossroad crashes. Characterization of SLA in patients with a decline in brain function appears to be an issue requiring urgent clarification. To take more effective countermeasures against traffic crashes, we have to adequately consider individual differences that remain unexplained with regard to age, particularly among elderly drivers. Detection of brain tissue changes by MRI may be a useful tool for addressing these differences and taking appropriate countermeasures.

## References

[1] DECADE OF ACTION FOR ROAD SAFETY 2011–2020. Available from; www.who.int/roadsafety/decade_of_action/en/ Accessed 2013 Jan 26.

[2] World Health Organization. World report on road traffic injury prevention. Geneva: World Health Organization, 2004.

[3] Hachinski VC, Potter P, Merskey H (1987) Leuko-araiosis. Arch Neurol 44: 21–23. 3800716 10.1001/archneur.1987.00520130013009

[4] Moody DM, Thore CR, Anstrom JA, Challa VR, Langefeld CD, et al (2004) Quantification of afferent vessels shows reduced brain vascular density in subjects with leukoaraiosis. Radiology 233: 883–890. 15564412 10.1148/radiol.2333020981

[5] Shinohara Y, Tohgi H, Hirai S, Terashi A, Fukuuchi Y, et al (2007) Effect of the Ca antagonist nilvadipine on stroke occurrence or recurrence and extension of asymptomatic cerebral infarction in hypertensive patients with or without history of stroke (PICA Study) 1. Design and results at enrollment. Cerebrovascular Disease 24: 202–209. 10.1159/000104478 17596689

[6] Park K, Yasuda N, Toyonaga S, Yamada SM, Nakabayashi H, et al (2007) Significant association between leukoaraiosis and metabolic syndrome in healthy subjects. Neurology 69: 974–978. 17538033 10.1212/01.wnl.0000266562.54684.bf

[7] Bokura H, Yamaguchi S, Iijima K, Nagai A, Oguro H (2008) Metabolic syndrome is associated with silent ischemic brain lesions. Stroke 39: 1607–1609. 18323475 10.1161/STROKEAHA.107.508630

[8] Sasaki M, Hirai T, Taoka T, Higano S, Wakabayashi C, et al (2008) Discriminating between silent cerebral infarction and deep white matter hyperintensity using combinations of three types of magnetic resonance images: a multicenter observer performance study. Neuroradiology 249: 624–630. 10.1007/s00234-008-0406-6 18551287

[9] Agresti A. Categorical Data Analysis. 2nd edition. New York: Wiley

[10] Garde E, Mortensen EL, Krabbe K, Rostrup E, Larsson HB (2000) Relation between age-related decline in intelligence and cerebral white matter hyperintensities in healthy octogenarians: a longitudinal study. Lancet 356: 628–634. 10968435 10.1016/S0140-6736(00)02604-0

[11] Van den Heuvel DM, ten Dam VH, de Craen AJ, Admiraal-Behloul F, Olofsen H, et al (2006) Increase in periventricular white matter hyperintensities parallels decline in mental processing speed in a non-demented elderly population. J Neurol Neurosurg Psychiatry 77: 149–153. 16421114 10.1136/jnnp.2005.070193 PMC2077562

[12] Debette S, Markus HS (2010) The clinical importance of white matter hyperintensities on brain magnetic resonance imaging: systematic review and meta-analysis. BMJ 341: c3666 doi: 10.1136/bmj.c3666. 20660506 10.1136/bmj.c3666 PMC2910261

[13] Debette S, Beiser A, DeCarli C, Au R, Himali JJ, et al (2010) Association of MRI markers of vascular brain injury with incident stroke, mild cognitive impairment, dementia and mortality: the Framingham Offspring Study. Stroke 41: 600–606. 20167919 10.1161/STROKEAHA.109.570044 PMC2847685

[14] Schmidt R, Petrovic K, Ropele S, Enzinger C, Fazekas F (2007) Progression of leukoaraiosis and cognition. Stroke 38: 2619–2625. 17673724 10.1161/STROKEAHA.107.489112

[15] Chabriat H, Pappata S, Poupon C, Clark CA, Vahedi K, et al (1999) Clinical severity in CADASIL related to ultrastructural damage in white matter. In vivo study with diffusion tensor MRI. Stroke 30: 2637–2643. 10582990 10.1161/01.str.30.12.2637

[16] O'Sullivan M, Summers PE, Jones DK, Jarosz JM, Williams SCR, et al (2001) Normal-appearing white matter in ischemic leukoaraiosis: a diffusion tensor MRI study. Neurology 57: 2307–2310. 11756617 10.1212/wnl.57.12.2307

[17] Maltz M, Shinar D (1999) Eye movements of younger and older drivers. Human Factors: The Journal of the Human Factors and Ergonomics Society 41: 15–25. 10.1518/001872099779577282 10354803

[18] Bao S, Boyle LN (2009) Age-related differences in visual scanning at median divided highway intersections in rural areas. Accident Analysis and Prevention 41: 146–152. 19114149 10.1016/j.aap.2008.10.007

[19] Brodal P. The Central Nervous System, Structure and Function. 2nd ed. New York: Oxford University Press, 1998.

[20] Filley CM (1998) The behavioral neurology of cerebral white matter. Neurology 50: 1535–1540. 9633691 10.1212/wnl.50.6.1535

[21] De Groot JC, de Leeuw FE, Oudkerk M, FRCPE JVG, Hofman A, et al (2002) Periventricular cerebral white matter lesions predict rate of cognitive decline. Ann Neurol 52: 335–341. 12205646 10.1002/ana.10294

[22] Prins ND, van Dijk EJ, den Heijer T, Vermeer SE, Koudstaal PJ, et al (2004) Cerebral white matter lesions and the risk of dementia. Arch Neurol 61: 1531–1534. 15477506 10.1001/archneur.61.10.1531

